# Functional Performance Testing and Patient Reported Outcomes following ACL Reconstruction: A Systematic Scoping Review

**DOI:** 10.1155/2014/613034

**Published:** 2014-11-16

**Authors:** Adel Almangoush, Lee Herrington

**Affiliations:** College of Health, Sport and Rehabilitation Sciences, University of Salford, Salford M6 6PU, UK

## Abstract

*Objective*. A systematic scoping review of the literature to identify functional performance tests and patient reported outcomes for patients who undergo anterior cruciate ligament (ACL) reconstruction and rehabilitation that are used in clinical practice and research during the last decade. *Methods*. A literature search was conducted. Electronic databases used included Medline, PubMed, Cochrane Library, EMBASE, CINAHL, SPORTDiscus, PEDro, and AMED. The inclusion criteria were English language, publication between April 2004 and April 2014, and primary ACL reconstruction with objective and/or subjective outcomes used. Two authors screened the selected papers for title, abstract, and full-text in accordance with predefined inclusion and exclusion criteria. The methodological quality of all papers was assessed by a checklist of the Critical Appraisal Skills Programme (CASP). *Results*. A total of 16 papers were included with full-text. Different authors used different study designs for functional performance testing which led to different outcomes that could not be compared. All papers used a measurement for quantity of functional performance except one study which used both quantity and quality outcomes. Several functional performance tests and patient reported outcomes were identified in this review. *Conclusion*. No extensive research has been carried out over the past 10 years to measure the quality of functional performance testing and control stability of patients following ACL reconstruction. However this study found that the measurement of functional performance following ACL reconstruction consisting of a one-leg hop for a set distance or a combination of different hops using limb symmetry index (LSI) was a main outcome parameter of several studies. A more extensive series of tests is suggested to measure both the quantitative and qualitative aspects of functional performance after the ACL reconstruction. The KOOS and the IKDC questionnaires are both measures that are increasingly being used for ACL reconstruction throughout the last decade.

## 1. Introduction

Reconstruction surgery is very common to restore a rupture of an anterior cruciate ligament (ACL). There is currently a multiplicity of functional performance tests and patient reported outcome measures to determine the success of this surgery and rehabilitation [1–4]. For instance, the review done by Garratt et al. found more than 15 patient-assessed health instruments specific to the knee in the 31 studies that were included [5]. Also, Wang et al. identified twenty-four unique instrument outcomes measurements for the knee [6]. Regarding functional performance tests the review done by Clark reported that more than 18 tests were used to evaluate the function of lower extremity following an ACL deficiency or ACL reconstruction [7]. In light of the abundance of tests available, there appears no consensus regarding which test or combination of tests is most appropriate for evaluating recovery following ACL reconstruction [2]. It has been recommended that a multiplicity of assessments, incorporating both functional performance testing and patient reported tools, is important to evaluate functional ability and outcome for patients following ACL reconstruction [2], but which of these tests or combination of tests provides the most rigorous test for outcome remains unclear. As no single instrument or functional performance test is currently capable of measuring all the multitude of parameters believed to relate to outcome, it is rational to accept that a range of tests should be administered to facilitate a full comprehensive evaluation of outcome.

Functional performance testing is likely to indicate the outcome of the neuromuscular training and appears to consist of two components [8]. The first component is the quantity of movement or the capabilities of the production of the force, for example, muscle strength measurements and hop tests [8]. The second component is the quality of movement, for example, the total knee flexion when landing from a jump or the occurrence of dynamic knee valgus [9, 10]. These two components are important in rehabilitation and prevention of ACL recurrent injuries or surgery failure [8, 11, 12]. Most papers describing the functional performance following ACL reconstruction are using the limb symmetry index (LSI) and thus are limited to quantitative measurements [13, 14]. Functional performance testing using qualitative methods evaluates compensation, or asymmetry, through clinical observation [15].

The limb symmetry index (LSI) calculation is commonly used when reporting the results of functional hop tests. The LSI is the percentage deficit of the distance hopped on the involved leg compared with the contralateral noninvolved leg [7]. The use of the LSI minimizes the probable confounding variable of the biological variation between people, which may affects the results [16]. The work of Munro and Herrington [17] showed LSI needs to be in excess of 90% to be deemed normal.

A functional outcome is a predicted result of care that is meaningful and practical for the patients and sustainable beyond the rehabilitation environment [18]. Functional outcomes not only assess benefits but also provide cost-benefit data. There are advantages and limitations to each measure used independently or in conjunction with other measures [18]. The practicality of functional outcome measures employed in the clinical/research setting is an important consideration [18]. Functional or performance tests provide an objective assessment of components of the patients' ability in a structured, controlled setting. Combining several tests to assess function may serve to minimize any trade-offs between specificity and sensitivity [19].

Regardless of which tests are selected, it is imperative that they be standardised, reliable, valid, and responsive to change with time as well as being clinically relevant [20–22]. Ideally, outcome measures in research and clinical practice should be low-cost, take an acceptable length of time to administer, be convenient for researcher and clinicians to use, and be acceptable to the participants under investigation [21, 22]. Therefore, the purpose of this scoping review was to identify and explore a number of commonly used outcome measures for patients following ACL reconstruction and postoperative rehabilitation to assess both aspects (quantitative and qualitative) of functional performance tests and self-reported questionnaires that have been used in last decade.

## 2. Methodology

We adopted a “systematic” scoping review approach—this is a combination of a scoping review methodology—to ensure the inclusion of broad areas of research and study designs and a systematic review of the methodology of the reviews [39]. A scoping review is a relatively new type of study providing an assessment of available evidence from the literature in a broad area of research such as the compliance in the reporting of clinical studies to established guidelines. It also serves to identify information gaps in the field and provide recommendations for implementation [39].

The methodology of scoping reviews was first described in detail by Arksey and O'Malley [39] in their pivotal paper published in 2005, which provided the foundation for carrying out a scoping review. This framework was further refined, and five stages were proposed to be followed when conducting a scoping review, including (1) the identification of a research question; (2) finding the relevant studies; (3) the selection of studies to be included in the review; (4) data extraction from the included studies; and (5) assembling, summarizing, and reporting the results of the review [40].

### 2.1. Search Strategy

A PRISMA compliant search strategy was used for study selection. The inclusion criteria of studies were as follows: (1) at least one lower extremity/knee functional performance test used as an outcome measurement of the article and/or patient reported outcomes, (2) subjects who were post-ACL reconstruction, (3) studies which were either randomised control trial (RCT), cross-sectional, or cohort designs, and (4) studies published in English between April 2004 and April 2014.

The electronic databases used were MEDLINE (MeSH terms), PubMed, Cochrane Library (systematic reviews and controlled trials registers), EMBASE, CINAHL, SPORTDiscus, PEDro (Physiotherapy Evidence database), and AMED (Allied and Complementary Medicine Index). In order to capture as many relevant references as possible, an expanded search was performed, including hand-searching the reference lists of all relevant articles, texts, and systematic reviews.

Search was conducted using the terms “knee” AND “ACL injuries” OR “functional performance” AND “measure” OR “test” OR “screen” OR “assessment” Or “patient reported.” The keyword search was also performed on PubMed utilising the key terms “anterior cruciate ligament” AND “surgery” AND “injury” AND “physical performance outcome measurements” to ensure a detailed and comprehensive search strategy, and the additional search was performed in academic textbook that contained an extensive review of functional performance tests [41] (see Table 1).

### 2.2. Study Identification

Two reviewers (Adel Almangoush and Lee Herrington) independently reviewed all titles and abstracts that were identified from the search strategy. In accordance with the predefined eligibility criteria the full-text manuscripts for all potentially eligible studies were obtained, and then in accordance with the predefined eligibility criteria the reviewers independently reviewed them a second time.

### 2.3. Data Extraction

Data extraction for each eligible paper was performed independently by two reviewers (Adel Almangoush and Lee Herrington) using a predefined spreadsheet. The reviewers' spreadsheets were amalgamated to create an agreed extraction form. The standardised data extraction form included details on (a) focus of study, study design, participant details, outcome measure (functional performance tests and patient reported outcomes), and results. In cases where insufficient data were provided within the publication, attempts were made to contact all corresponding authors to identify such data.

### 2.4. Critical Appraisal

Each study's methodological quality was assessed by using an appraisal tool devised to specifically evaluate functional performance testing and patients' reported questionnaires of studies that included those patients following ACL reconstruction. This was based on the Critical Appraisal Skills Programme (CASP) critical appraisal tool (CASP, 2007) [42], which has been widely used and employed in previous systematic reviews to evaluate the methodological quality of clinical studies [43–45]. The tool assessed domains such as the identification of the research questions, appropriateness of the research design, surgery and rehabilitation outcomes, the accuracy of description of methodology and population, appropriateness of analysis methods, and interpretation of findings. The appraisal was independently undertaken by two aforementioned reviewers (Adel Almangoush and Lee Herrington). If any disagreements arose regarding the study selection, data extraction, or appraisal score, these were sorted out through discussion between the two reviewers until a consensus was met. Studies were excluded if they achieved a very low methodological score of less than 50% through the CASP scoring system. A total score was calculated by adding up all positive items.

### 2.5. Data Analysis

All analyses were initially undertaken by one reviewer (Adel Almangoush) and verified by the other reviewer (Lee Herrington). A narrative review was undertaken of all included literature. An assessment of the quantity and quality of functional performance testing and patient reported tools of those patients following ACL reconstruction by means of a meta-analysis was planned. However, unfortunately due to the heterogeneity of the studies, in particular the information regarding surgery and rehabilitation outcomes, it was not possible to complete this analysis.

## 3. Results

### 3.1. Search Strategy

**Figure 1:**
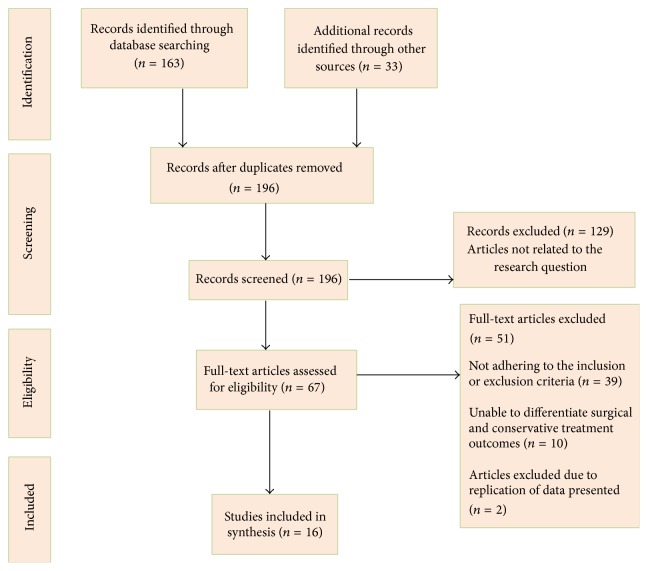
PRISMA follow diagram to depict search strategy results.

A PRISMA compliant search strategy was used, and results are presented in a PRISMA flow diagram (Figure 1) [46]. As Figure 1 demonstrates, a total of 196 citations were identified through the search strategy. Sixteen papers satisfied the eligibility criteria and were therefore included in the review. This included 10 randomised controlled trials and 6 cohort studies. These were summarised in Table 2.

### 3.2. Knee Laxity

Eleven studies assessed knee laxity using a variety of instrumented laxity tests. Nine studies used a KT-1000 arthrometer (MEDmetric, San Diego, CA, USA) [26, 27, 29, 31, 33–37]. One study used a manual maximum test with a Rolimeter (Aircast, Summit, NJ, USA) [32]. One study used Lachman test and/or pivot shift test [30]. All studies assessed the anterior displacement of the tibia relative to the femur, except one study which used medial joint space opening on manual valgus stress testing [32].

#### 3.2.1. Critical Appraisal

The findings of the critical appraisal are summarised in Table 3. On analysis, the literature presented with a number of methodological limitations. Only six papers (38%) justified their sample sizes based on power calculations. Whilst the surgery management strategies undertaken were clearly described in most of these papers, only four publications presented sufficient information to reproduce their methodologies for physiotherapy treatments and described the rehabilitation programs undertaken (25%). Furthermore, whilst all studies reviewed used appropriate outcome measures to evaluate their participants, only a few of them defined the presence of an observer. Whilst inferential statistics were presented in all included publications, confidence intervals were only provided in four papers (25%). No study presented a standard error of measurement. None of the included studies evaluated the patients before the ACL operation. However, all authors interpreted their findings appropriately and related these results in a suitable manner to clinical practice and the existing evidence base. All papers passed more than 50%.

#### 3.2.2. Study Description

*Outcome Measures*. A variety of different functional performance tests and patient reported outcomes measures have been reported in patients following ACL reconstruction. These were assessed individually as shown below.

### 3.3. Functional Performance Testing

#### 3.3.1. Hop Tests

A number of different assessment methods were used to determine the functional performance of patients following ACL reconstruction. These methods included the one-leg hope for distance; this is a commonly used functional performance test of both strength and confidence in the tested leg; it correlates positively with muscle strength and power [7, 47]. The one-leg hope for distance was assessed in fourteen studies (88%) of the papers included. Triple hop test for distance was evaluated in four papers [31, 32, 34, 37]. Three studies described a 6-meter timed hop test for speed [31, 32, 34]; crossover hop of distance was assessed in two studies [31, 34]; side hop and vertical jump were also assessed in two studies [21, 38]; triple-jump test and stair hop test were evaluated in one study only [35]; and functional squat test was assessed in only one study also [24]. More than 50% of studies used the hop tests as a measurement of function within the battery of different tests completed. Only seven studies used multiple hop tests (44%), and only seven papers (less than 50%) reported limb symmetry index (LSI) comparing the injured with uninjured leg. Only one study described the quality of movement whilst carrying out the test (e.g., dynamic knee valgus or knee flexion angle) [38].

### 3.4. Postural Control

Postural stability of patients following ACL reconstruction was assessed in four studies by using different measurement methods. Baltaci et al. and Delahunt et al. used the modified star excursion balance test (SEBT) to evaluate the postural control of their patients [24, 28]. Risberg et al. [35] and Zouita Ben Moussa et al. [25] used the NeuroCom Balance Master platform system to measure the postural stability. Balance was recorded using static and dynamic balance tests on an instrumented unstable platform (KAT2000).

### 3.5. Patient Reported Outcomes

Several reported questionnaires presented in the papers were evaluated in this scoping review, whereas KOOS and IKDC were assessed in most of the selected papers. Only four studies used Lysholm score [29, 32, 33, 36], three papers assessed the Tegner activity level rating scale [23, 27, 32], and only two studies per each score evaluated the global rating scale [31, 34], the KOS-ADLS questionnaire [31, 34], and the Cincinnati knee score [35, 37].

Kocher et al. [48] made a comprehensive analysis of determinants of patient reported outcomes after ACL reconstruction. They concluded that subjective variables are more important for evaluation of patient reported outcomes than objective findings. They found 7 “key” symptoms that together accounted for 83% of the variability in patients reported outcomes.

## 4. Discussion

The authors of the current review aimed to identify existing functional performance testing and patient reported outcomes for patients following ACL reconstruction in the last decade. The most important finding of the present study was that all included articles used limited quantitative measurements to determine functional performance, except the study done by Trulsson et al. [38]. In the last decade most of the studies included in this review were focusing on the hop tests especially the single-leg hop test and few of these studies looked at a postural stability. Regarding the reported outcomes the focus was on the KOOS and IKDC questionnaires.

### 4.1. Functional Performance Testing

Although the included articles reported the use of several hop tests, fourteen studies used a single-leg hop for distance as the gold standard for measuring functional performance after ACL reconstruction because the reliability of this test is high (ICC ranging from 0.86 to 0.95) [49, 50]. The relative reliability of the single hop for distance test in patients 1 to 2 years following ACL reconstruction has previously been reported [51]. However, several studies showed that the sensitivity increases when two or more different hop tests are performed [50–52]. By using multiple hop tests, their qualities can be assessed and thereby the opportunity to detect discrepancies in hop performance increases [49]. There is a strong relationship between crossover hop performance and functional outcome [38] correlating significantly to IKDC subjective and KOOS questionnaire scores [53]. The most reliable and valid of the multitude of hop tests in relation to the ACLR patient would appear to be the single hop for distance and the crossover hop tests [7, 50, 54]. The ability of the ACLR patient to perform well during hop tests is of paramount importance when judging functional performance.

Hop testing has frequently been proposed as a practical performance-based outcome measure that reflects the integrated effect of neuromuscular control, strength (force-generating capacity), and confidence in the limb and requires minimal equipment and time to administer [55]. Based on a review of the potential use of hop tests as measures of dynamic knee stability, Fitzgerald et al. [56] suggested that hopping may be appropriate for use as a predictive tool for identifying patients who may have future problems as a result of knee injury or pathology and as an evaluative tool to reflect change in the patient status in response to treatment.

Within the published literature, the “gold standard” is often regarded as having a limb symmetry index (LSI) of greater than 85% [7], indicating that anything less than a 15% deficit in strength between the operated and nonoperated limb is acceptable. This works on the assumption that the uninjured limb is “normal” in terms of its strength [7]. A study conducted in [57] has shown that the contralateral (noninjured) leg is significantly weaker than matched controls. Therefore, this assumption of normality should be viewed with caution, as the period of time during both preoperative and postoperative rehabilitation is likely to have caused atrophy of the noninjured leg. However, using the LSI is debatable because recent studies have shown that an ACL injury could lead to a crossover effect in the uninvolved leg resulting in strength and function loss based on biomechanical and neuromuscular changes [16].

### 4.2. Postural Control

To the best of the knowledge of this study's researchers, there are few published studies that search for postural stability following ACL reconstruction [58]. For example, the SEBT outcome measure offers a simple, reliable, valid, and low-cost alternative to more sophisticated instrumented methods, to assess dynamic balance ability [59, 60]; unlike force plates or electronically controlled balance platforms, it is an easy and highly portable test that could be employed in a range of clinical environments. According to Logerstedt et al. [54] the grid required testing for ACL deficiency patients, three lines are positioned on the grid (anterior, medial, and lateral reach distance) which are labelled according to the direction of excursion relative to the stance leg.

High intertester reliability of the SEBT has previously been reported [59]. Whilst previous studies have evaluated intratester reliability [59], only one study has evaluated between-session reliability of the SEBT with normalised scores with ICC values ranging from 0.89 to 0.93 [61]. However, only 3 reach distances, anterior, posteromedial, and posterolateral, were evaluated. Therefore, further study of between-session reliability of all reach directions is warranted.

Previous research has suggested that the SEBT is reliable and sensitive enough to detect dynamic postural control deficits in patients with an ACL-deficient (ACL-D) limb [62, 63]. In these studies, patients who were injured were shown to have lower SEBT scores compared to those of their uninjured limb and those of healthy participants. In particular, Herrington et al. [62] found that patients with ACL deficiency showed functional deficits in the anterior, medial, lateral, and posteromedial reach directions.

Functional tests are a quick and inexpensive method of obtaining an objective measure of lower limb function following surgery [52]. These tests are thought to provide an indication of muscle strength and power, neuromuscular control and confidence [64, 65]. Additionally, a number of authors have highlighted that a single functional test may not be sensitive enough to detect performance limitations and that at least two functional tests should be used [51, 52, 55].

Furthermore, all included studies reported quantitative data such as distance and/or time. Only one study described the quality of movement whilst carrying out the test (e.g., dynamic knee valgus or knee flexion angle) [38]. Studies focusing on prevention showed that the risk for ACL injuries was reduced when training was done before high quality trials [66–68]. For ACL injury screening, Ekegren and his colleagues examined dynamic knee valgus during a drop-jump task. The drop jump turned out to be a reliable and valid instrument in observing the dynamic knee valgus [9]. von Porat et al. investigated videotaped functional performance tests in ACL injured subjects, and they reported that observation is a reliable and valid instrument for assessing knee flexion angles of the one-leg hop for a distance [10]. The single-leg squat (SLS) test is a cost-effective and simple movement to determine lower extremity alignment in the coronal plane. Carried out with a single camera in any setting, this procedure can visibly identify a valgus lower extremity alignment on landing, which is considered to be a potential risk factor for a possible noncontact ACL injury [12]. The SLS test has been described in a number of studies as a useful clinical measure to identify hip muscle function and dynamic knee control [69].

### 4.3. Patient Reported Outcomes

Patient reported instruments are normally related to signs and symptoms experienced by the patient and/or the functional tasks that individuals are able to achieve during their activities of daily living [65]. A commonly used knee outcome instrument is the Cincinnati knee scoring scale, and although it has been demonstrated to be an adequate tool to evaluate knee function following ACL reconstruction [70], it also includes manual and instrumented stability testing to assess symptoms and function; thus it becomes more difficult to separate various aspects of knee function following ACL injury.

The International Knee Documentation Committee (IKDC) developed a scoring system for knees with ACL injuries. The IKDC is reliable and the validity and responsiveness were found to be good [71]. The IKDC, the Cincinnati knee scoring scale, and the first version of the Lysholm score are assessor reported scores, which have been exposed to be biased when applied to individuals with an ACL injury [72]. On the other hand, the Lysholm-Tegner system is much simpler but mainly evaluates symptoms and activity. Carlos argued that for those clinicians and researchers considering using only the IKDC as their patient reported outcomes for ACL reconstruction, they should include as a minimum the KOOS subscales that address broader areas of concern, including quality of life and emotional health that are most important to patients following ACL reconstruction and are not wholly represented in IKDC [73]. Moreover, there is a suggestion that the KOOS is perhaps more suitable for the assessment of patients in the longer term unlike the IKDC [74]. The KOOS has shown good validity and demonstrated that it is responsive to ACL reconstruction and rehabilitation; it shows that it is a reliable instrument for patients undergoing ACL surgery and rehabilitation [74]. KOOS has been used in an extensive amount of current research protocols and it has been translated and culturally adapted into various languages [75]. Clinicians and researchers looking to use a patient-based score measure of outcomes must consider the specific patient population in which it has been evaluated. Using a diagnostic algorithm that measures the anatomic parts of the knee as separate constructs may solve this dilemma, allowing for the measurement of treatment outcomes across patient groups and the selection of the optimal clinical intervention.

In general, the papers in this literature review included poorly described sample sizes and whether or not the sample size was based on power calculations. Accordingly, the samples recruited may not necessarily have been big enough to identify a difference in outcome following a rehabilitation programme, irrespective of whether or not a difference existed [76]. The papers weakly described who had assessed the subjects. Accordingly, it was not possible to determine whether measurement error influenced the results obtained or whether the experiences or training of the assessors was a variable which may have accounted for any between-study differences.

*Limitations*. There are limitations of this systematic scoping review that should be acknowledged. For instance, the authors established very specific inclusion/exclusion criteria for selection of functional performance tests included inthis review. This included only the functional performance tests for ACL reconstruction patients after surgery. Many tests were excluded because the studies were performed on healthy people or subjects with various neurological or debilitating comorbidities. Therefore, it is possible that some functional performance tests were not identified. This may modify the interpretation of the values attained for a specific functional performance test, and this was also the reason for the small number of studies included.

Future studies are required to establish the reliability and validity of existing functional performance tests or explore new, relevant quality measurements of the functional performance tests to be used in patients following ACL surgery.

## 5. Conclusion 

This review shows that, following the ACL reconstruction, the one-leg hop for distance or a combination of different hops and the limb symmetry index (LSI) of functional performance tests was used as a main outcome parameter of several studies. No extensive research has been carried out over the past 10 years to measure the control stability of patients following ACL reconstruction. Furthermore, no observation or videotaping was used to assess the quality of any test of any functional performance and control stability of ACL patients following surgery except for one study. Because previous studies discuss additional important parameters, a more extensive battery of tests is suggested to measure both the quantitative and qualitative aspects of functional performance after the ACL reconstruction. The KOOS and the IKDC are both measures that are increasingly being used for ACL reconstruction during the last 10 years.

## Figures and Tables

**Table 1:** Search terms adopted for a Medline search strategy.

Number	Search term
1	Functional
2	Performance
3	Measure
4	Screen
5	Assessment
6	Objective
7	Subjective
8	Questionnaire
9	Surgery
10	ACL
11	Knee
12	Injury
13	Anterior cruciate ligament
14	OR/6-8
15	OR/10-12
16	OR/9-10
17	OR/1-2-3
18	OR/1-4
19	OR/5-7

**Table 2:** Summary of the studies reviewed.

Eligible studies	Focus of study	Study design	Participant details: gender (F/M), subject (age)	Knee laxity	Functional performance tests		Patient reported tools	Results (LSI)
					Quantitative assessment	Qualitative assessment		
(1) Ageberg et al. [23]	Investigation of functional performance for ACLR patients at 2–5 years after injury.	Cohort study	54 patients (ages 18–35 years)	N/A	One-leg hop for distance, vertical jump, side hop	N/A	KOOS, Tegner	One-leg hop for distance LSI 99.5%, vertical jump LSI 96.4%, side hop LSI 97.9

(2) Baltaci et al. [24]	Determination of acceptability of a Nintendo Wii Fit compared to conventional rehabilitation as a therapy tool for ACLR patients.	RCT	30 men Wii Fit (*n* = 15; mean age, 29 ± 7 years) or conventional rehabilitation (*n* = 15; mean age, 29 ± 6 years)	N/A	Functional squat tests, SEBT	N/A	N/A	N/A

(3) Zouita Ben Moussa et al. [25]	‘‘To analyse postural stability and single-leg hop” measurements in post-ACLR subjects and compare them with an age- and activity-matched control group.	RCT	26 patients	N/A	Hop for distance, one-leg stance postural stability	N/A	N/A	N/A

(4) Beynnon et al. [26]	Investigation of any difference in the patient satisfaction and functional performance when providing rehabilitation with either an accelerated or nonaccelerated program.	RCT	22 patients 11 M/11 F	Kt-1000	One-leg hop for distance	N/A	KOOS, IKDC	N/A

(5) Beynnon et al. [27]	Investigation of any difference in patient satisfaction, functional performance, and activity level, between patients treated with accelerated versus nonaccelerated rehabilitation programs.	RCT	36 patients 22 M/14 F	Kt-1000	One-leg hop for distance	N/A	KOOS, IKDC, Tegner	N/A

(6) Delahunt et al. [28]	Investigation of dynamic postural stability as quantified by the SEBT and simultaneous hip and knee joint kinematics in participants with previous ACL reconstructions.	Cohort study	31 patients all female	N/A	SEBT	N/A	KOOS, IKDC	N/A

(7) Halinen et al. [29]	Determination of whether nonoperative or early operative treatments of grade III medial collateral ligament rupture lead to similar results.	RCT	47 patients 27 F/20 M	Kt-1000	One-leg hop for distance	N/A	IKDC, Lysholm score	N/A

(8) Halinen et al. [30]	Evaluation of the effect of early repair of the concomitant medial collateral ligament injury on the range of motion of the knee in ACLR patients.	RCT	47 patients	Lacham	One-leg hop for distance	**N/A**	N/A	One-leg hop for distance: at 52 weeks: Group I LSI 83.1%, Group II LSI 86.1%; at 104 weeks: Group I LSI 90.2%, Group II LSI 93.4%

(9) Hartigan et al. [31]	Determination of an effective intervention for improving readiness to return to presurgery activity.	RCT	40 patients 29 M/11 F (average age of 28.4 years)	Kt-1000	Single hop crossover hop, and triple hop tests for distance and the 6-meter timed hop test for speed	**N/A**	**N/A**	**6-meter timed hop test:** Group I LSI 89.2%, Group II LSI 89.8%; **One-leg hop test:** Group I LSI 83.7%, Group II LSI 83.1%; **Crossover hop test:** Group I LSI 81.7%, Group II LSI 85.6%; **Triple hop test:** Group I LSI 82.4%, Group II LSI 86.4%

(10) Lindström et al. [32]	Using computed tomography (CT) to analyze muscle cross-sectional area and attenuation ratios (operated/nonoperated knee).	Cohort study	37 patients 23 M/14 F (mean age 26.5 Years, range = 16–54)	Rolimeter	One-leg hop, triple hop, square hop, 6 m timed hop	N/A	KOOS, Lysholm, knee score Tegner, activity level rating scale	One-leg hop, preoperative LSI 0.82%, postoperative LSI 0.93%

(11) McDevitt et al. [33]	Determination of postoperative functional knee and its influences outcomes.	RCT	100 patients	Kt-1000	Single-legged hop test	**N/A**	IKDC, Lysholm scores	N/A

(12) Moksnes and Risberg [34]	Comparison of the functional outcome between ACLR and nonoperative treatment.	Cohort study	125 patients (ages between 14 and 60 years)	Kt-1000	One-leg hop test, the triple hop, the triple crossover hop, and the 6 m timed hop test	**N/A**	(KOS-ADLS), IKDC, global rating of knee function (VAS 0–100)	Single hop, LSI 91.8%, triple hop 91.4%, triple crossover hop LSI 93.5%, 6 m timed hop test LSI 94.2%

(13) Risberg et al. [35]	Determination of the effect of a 6-month neuromuscular training (NT) program versus a traditional strength training (ST) program following ACL surgery.	RCT	75 patients 27 F/47 M (mean age 28.4 years, range = 16.7–40.3)	Kt-1000	One-leg hop test, triple-jump test, and stair hop test; balance was recorded using static and dynamic balance tests	N/A	The Cincinnati knee score, two VASs were included: one for pain intensity and one for global knee function SF-36	**One-leg hop test:** preoperative: Group I LSI 93.7%, Group II 90.1%; at 6 months following surgery: Group I LSI 81.0%, Group II LSI 84.9% **Triple-jump test:** preoperative: Group I LSI 94.6%, Group II LSI 91.8%; at 6 months following surgery: Group I LSI 83.1% Group II LSI 88.5% **Stair hop test:** preoperative: Group I LSI 84.8%, Group II LSI7 8.4%; at 6 months following surgery: Group I LSI 79.8%, Group II LSI 79.8%

(14) Salmon et al. [36]	Determining if there is any difference in outcome between men and women after anterior cruciate ligament reconstruction.	Cohort study	200 patients 100 M/100 F	Kt-1000	Single-legged hop test, kneeling pain	**N/A**	IKDC, Lysholm knee score	N/A

(15) Shaw et al. [37]	The investigation of the effectiveness of quadriceps exercises following anterior cruciate ligament reconstruction.	RCT	103 patients 28 F/75 M	Kt-1000	Single-leg hop test, triple hop	N/A	Cincinnati knee rating system	Single-leg hop test, no quadriceps exercise LSI 81.7%, quadriceps exercise LSI 83.8% Triple hop, no quadriceps exercise LSI 81.8%, quadriceps exercise LSI 83.7%

(16) Trulsson et al. [38]	The correlation between a novel test set and commonly used tests of knee function.	Cohort study	53 patients (mean age 30 years, range 20–39)	N/A	vertical jump, the one-leg hop, and the side hop	Test for substitution patterns (TSP)	KOOS	N/A

**Table 3:** The Critical Appraisal Skills Program results (CASP).

The criteria	(1) Ageberg et al., 2008 [23]	(2) Baltaci et al., 2013 [24]	(3)Zouita Ben Moussa et al., 2009 [25]	(4) Beynnon et al., 2005 [26]	(5) Beynnon et al., 2011 [27]	(6) Delahunt et al., 2013 [28]	(7) Halinen et al., 2006 [29]	(8) Halinen et al., 2009 [30]	(9) Hartigan et al., 2010 [31]	(10) Lindström et al., 2013 [32]	(11) McDevitt et al., 2004 [33]	(12) Moksnes and Risberg, 2009 [34]	(13) Risberg et al., 2007 [35]	(14) Salmon et al., 2006 [36]	(15) Shaw et al., 2005 [37]	(16) Trulsson et al., 2010 [38]
Study design	N	Y	N	Y	Y	Y	Y	N	Y	Y	Y	Y	Y	Y	Y	Y
Focused question	Y	Y	Y	Y	Y	Y	Y	Y	Y	Y	Y	Y	Y	Y	Y	Y
Appropriate design	Y	Y	Y	Y	Y	Y	Y	Y	Y	Y	Y	Y	Y	Y	Y	Y
Population defined	Y	Y	Y	Y	Y	Y	Y	Y	Y	Y	Y	Y	Y	Y	Y	Y
Recruitment methods acknowledged	Y	Y	Y	Y	Y	N	Y	N	Y	Y	Y	Y	Y	Y	Y	Y
Sample size defined by power	Y	N	N	N	Y	N	N	Y	Y	Y	N	N	Y	Y	N	N
Study setting described	Y	Y	Y	Y	Y	Y	Y	Y	Y	Y	Y	Y	Y	Y	Y	N
Interventions described (surgery)	Y	Y	Y	Y	Y	N	Y	Y	Y	Y	Y	Y	Y	Y	Y	N
Rehabilitation described	N	Y	N	Y	N	N	N	N	N	Y	N	N	Y	N	Y	N
Outcome measures defined	Y	Y	Y	Y	Y	Y	Y	Y	Y	Y	Y	Y	Y	Y	Y	Y
Observers defined	Y	N	N	Y	Y	Y	N	N	Y	Y	Y	Y	Y	Y	Y	Y
Statistical methods described	Y	Y	Y	Y	Y	Y	Y	Y	Y	Y	Y	Y	Y	Y	Y	Y
Variance described	N	N	N	N	N	N	N	N	N	N	N	N	N	N	N	N
Inferential statistics employed	Y	Y	Y	Y	Y	Y	Y	Y	Y	Y	Y	Y	Y	Y	Y	Y
Confidence intervals presented	Y	N	N	N	N	Y	N	N	N	N	N	Y	N	Y	N	N
Appropriate interpretation	Y	Y	Y	Y	Y	Y	Y	Y	Y	Y	Y	Y	Y	Y	Y	Y
Generalizability	Y	Y	Y	Y	Y	Y	N	N	Y	Y	Y	Y	Y	Y	Y	Y
Relevance to present evidence base	Y	Y	Y	Y	Y	Y	Y	Y	Y	Y	Y	Y	Y	Y	Y	Y
Clinical relevance discussed	Y	Y	Y	Y	Y	Y	Y	Y	Y	Y	Y	Y	Y	Y	Y	Y

The score	16	15	13	16	16	14	13	12	16	17	15	16	17	17	16	13
